# Novel bioactive compounds from marine-derived endosymbiotic Penicillium fungi reported during 2020–2025

**DOI:** 10.1007/s13659-026-00633-z

**Published:** 2026-06-23

**Authors:** Sarah A. Omran, Mohamed S. Elnaggar, Fadia S. Youssef, Safaa A. El-Moghazy

**Affiliations:** 1 Department of Pharmacognosy, Faculty of Pharmacy, Badr University in Cairo (BUC), Cairo, Egypt; 2 Department of Pharmacognosy, Faculty of Pharmacy, Ain Shams University, 11566 Cairo, Egypt

**Keywords:** Marine-derived *Penicillium*, Natural products, MNPs, Antimicrobial, Cytotoxic, Anti-inflammatory

## Abstract

Marine-derived *Penicillium* fungus gained significant attention in recent years due to their potential as a valuable source of unique natural compounds with a wide range of chemical structures and bioactive capabilities. The primary origins of marine-derived *Penicillium* fungi are mangroves, sediments, marine shrimps, corals, starfish, algae, and sponges. A total of 390 unique natural compounds were extracted from *Penicillium* fungi which were found in marine environments. These compounds primarily consist of polyketides, alkaloids, terpenoids, chromone derivatives, anthraquinones, citrinins, steroids, azaphilones, and macrolides. Biological studies demonstrated that these compounds exhibit antimicrobial, anti-inflammatory, cytotoxic, pesticide development, neuroprotective, anti-glioma, anti-proliferative, α-glucosidase inhibitory activities, anti-allergic, photo-protective, anti-angiogenic effect, larvicidal activity, antiviral, anti-diabetic activity, and other activities that could be useful in the development of new drugs. This study shed the light on the recent discovery of bioactive MNPs (marine-derived *Penicillium*; natural products) derived from *Penicillium* fungi found in marine environments in the last five years from 2020 to 2025, classified the MNPs based on the sources of the fungi and their specific biological activities. Besides, the results of molecular docking studies recently performed on *Penicillium* metabolites referring to various biological activities were also compiled in this review. Thus, this review highlighted that *Penicillium* species obtained from marine organisms act as an everlasting mine of bioactive novel metabolites combating various ailments.

**Graphical Abstract:**

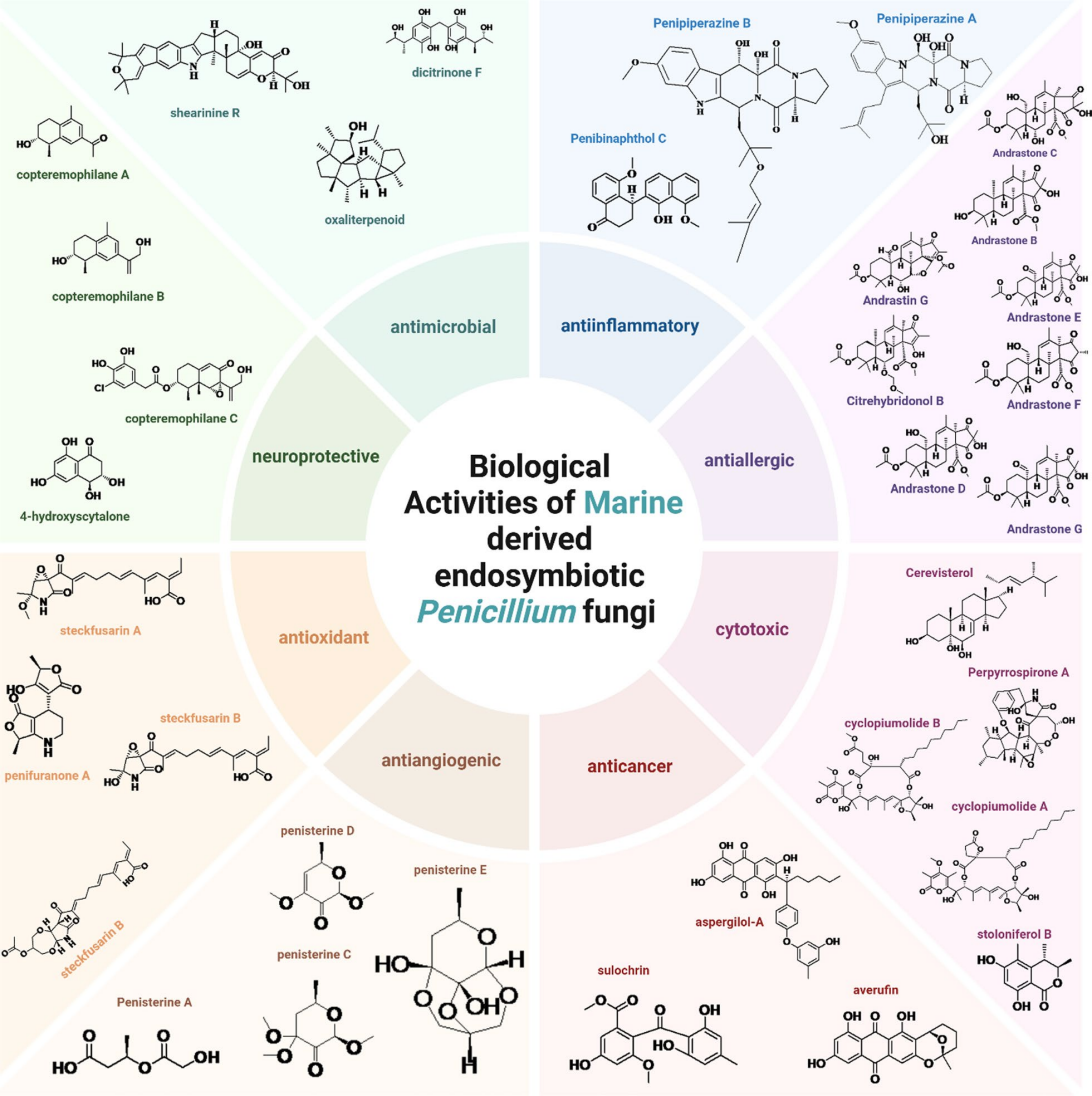

## Introduction

The oceans, which cover over 70% of the Earth's surface, undeniably provide extensive ecosystems and serve as abundant suppliers of diverse living organisms. The marine environment is distinct from the terrestrial environment due to its unique characteristics, such as high pressure, high salt content, extremely cold temperatures, and limited availability of nutrients. The distinctive metabolic and adaptation mechanisms of marine microorganisms give rise to their ability to produce a wide range of structurally and functionally diverse natural products (NPs) [1, 2].

Marine microorganisms must endure a challenging environment characterized by extreme conditions such as high pressure (up to 1100 atmospheres), lack of oxygen (anaerobic conditions), sub-zero temperatures, highly acidic conditions (pH 2.8), and extremely high temperatures (over 100°C) in hot springs. Furthermore, it was important to acclimatize to elevated salinity, radiation, luminosity, and diminished nutrient levels. Under such circumstances, the harsh environment facilitated genetic and metabolic variation in marine microbes, resulting in distinct adaptive mechanisms, particularly the production of uncommon defensive substances [3–5].

Recent research demonstrated that marine microorganisms showed the ability to produce a wide range of distinct metabolites that possess diverse biological characteristics. These metabolites are anticipated to have applications in the pharmaceutical, cosmetic, and medical sectors [3, 6]. Marine fungi are a group of organisms that have a wide range of biochemical characteristics and are a potentially valuable source of new bioactive natural chemicals. Marine fungi generate a variety of secondary metabolites, including as terpenes, steroids, polyketides, peptides, alkaloids, and polysaccharides [5, 7, 8].

The primary associations of these metabolites are with antibacterial, anticancer, antiviral, antioxidant, and anti-inflammatory properties. Given the diverse spectrum of actions, these metabolites show significant potential for drug development and various uses in the fields of medicine, pharmaceuticals, agriculture, and cosmetics [9, 10]. Marine fungi, specifically those belonging to the *Penicillium* and *Aspergillus* genera, are highly productive in synthesizing a wide range of natural substances that possess functional or medicinal qualities. The fungi were effectively isolated and identified from several marine sources, such as sponges, coral, algae, mangroves, sediment, and seawater [4, 11]. Marine fungi can be found in both deep and surface oceans and they are dependable supply of several beneficial molecules such as antimicrobials, antioxidants, and anticancer agents [3].

By the end of 2016, some 28,500 marine natural products, such as polysaccharides, peptides, polyketides, polyphenolic compounds, sterol-like products, and alkaloids, had been discovered. Marine natural compounds have a wide array of biological actions, encompassing anticancer, antibacterial, antifungal, and antiviral properties. The wide variety of activities exhibited by secondary metabolites makes them highly attractive for the development of new medication prototypes [3]. This study highlighted the recent discovery of bioactive MNPs (marine-derived *Penicillium*; natural products) derived from *Penicillium* fungi found in marine environments in the last five years from 2020 to 2025, classified the MNPs based on the sources of the fungi and their specific biological activities. Besides, the results of molecular docking studies recently performed on *Penicillium* metabolites referring to various biological activities were also compiled in this review.

## Structural classes of novel bioactive secondary metabolites from marine-derived endosymbiotic *Penicillium* fungi and their activities

### Alkaloids

**Fig. 1:**
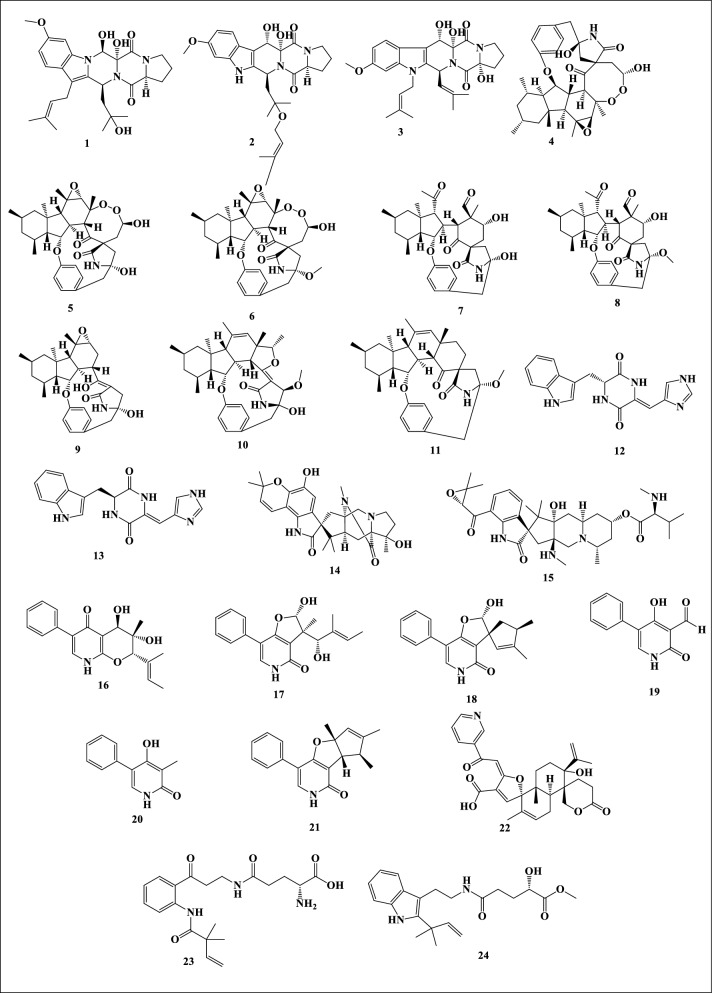
Novel alkaloids isolated from *Penicillium* genera associated with marine sources

Twenty-four alkaloids as presented in Fig. 1 of different sub classes were identified from *Penicillium* genera associated with marine sources. They are as follows.

#### Diketopiperazine alkaloids

Diketopiperazine alkaloids are a distinct group of nitrogen-containing chemicals that possess a diketopiperazine skeleton formed by the condensation of two amino acids. So far, several diketopiperazine alkaloids with promising antiviral, antibacterial, anticancer, and anti-inflammatory properties were identified in *Penicillium* fungi isolated from marine environments. Diketopiperazine alkaloids possess distinctive structures and demonstrate significant potential activity, making them highly promising subjects for further investigation [12]. The marine-derived fungus *Penicillium brasilianum* yielded two novel diketopiperazine alkaloids, namely penipiperazine A **(1)** and its biogenetically related new metabolite Penipiperazine B **(2)**. Compounds (**1)** and **(2)** effectively suppressed the expression of pro-inflammatory cytokines in RAW264.7 cells treated with LPS and the release of nitric oxide (NO). This indicates that they showed potential as promising candidates for the development of anti-inflammatory agents [13]. Several chromatographic techniques were employed to separate the ethyl acetate (EtOAc) extract from the fermentation cultures of the fungus strain TW58-16. Consequently, a novel diketopiperazine alkaloid, (8S,9R,12R,18S)-12-hydroxy-fumitremorgin B **(3)**, was produced and obtained from the marine-derived fungus *Penicillium* sp. TW58-16 [14].

#### Hirsutellone alkaloids

A new hirsutellone alkaloid with a unique structure consisting of a 6/5/6/8/5/13/6 oxahexacyclic scaffold and a peroxide-bridged 8,9-dioxa2-azaspiro[4.7]dodecane core, perpyrrospirone A **(4)** was isolated from the marine-derived *Penicillium citrinum*. Compound 4 demonstrated cytotoxic effects on various human tumor cell lines (HeLa, Bel-7402, MCF-7, MDA-MB-231, HepG2, and and MGC803) with IC_50_ values ranging from 9.1 to 38.9 μM [15].

#### Decahydrofluorene-class alkaloids

Seven novel decahydrofluorene-class alkaloids, namely pyrrospirones K-Q **(5–11)**, were extracted from marine-derived Fungus *Penicillium* sp. SCSIO 41512. From a biological perspective, compounds (**5)**, **(6),** and **(9)** exhibited antibacterial properties against all or part of the six pathogens: *E. coli*, *S. aureus*, *B. subtilis, S. aureus* MRSA, *S. agalactiae*, and *B. amyloliquefaciens*. Pyrrospirones **(8)** and **(10)** exhibited moderate inhibitory effects on several protein tyrosine phosphatases (PTPs), with IC_50_ values ranging from 39.4 to 100 µM [16].

#### Indole alkaloids

Marine-derived indole alkaloids are widely recognized to have a variety of biological functions in addition to unique structures [17]. In this regard, indole alkaloids 11R,14E-(+)- penilloid A **(12)** and 11S-( −)-penilloid A **(13)** were obtained from marine-associated *Penicillium* sp. ZZ1750. Although 11R,14E-(+)- penilloid A (13) and 11S-(−)-penilloid A (12) showed no antiglioma activity, it is necessary to do further evaluations on the additional functions of these recently discovered indole alkaloids [18]. Another study revealed that the fungus *Penicillium* janthinellum HK1-6, which was obtained from the rhizosphere soil of a mangrove, yielded a novel prenylated indole alkaloid, designated as paraherquamide J **(14)** [19]. Besides, Jiang et al. showed in their study that citrinadin C **(15)**, a novel pentacyclic spirooxindole alkaloid, was obtained from deep sea sediment-derived *Penicillium citrinum* and exhibited cytotoxic effects on the MHCC97H human liver cancer cell line, with an IC_50_ value of 16.7 μM [20].

#### Pyridone alkaloids

Pyridones are a distinctive category of heterocycles that contain a carbonyl group and a nitrogen heteroatom in a 6-membered aromatic ring. There are two forms of pyridones that are distinguished based on the position of the nitrogen atom relative to the carbonyl group which are 2-pyridones and 4-pyridones [21]. Yan et al. conducted a study showing that the marine-derived *Penicillium* sp*.* XZD3-3, found in the intestine of a marine shrimp, yielded six recently discovered pyridone alkaloids which are citridones H–L **(16–20)** and ent-citridone A **(21).** Compounds **(17)** and **(18)** exhibited moderate inhibition against nitric oxide production, with IC_50_ values of 52.5 µM and 81.6 µM, respectively, compared to positive control hydrocortisone with an IC_50_ value of 22.4 µM [22].

#### Meroterpenoid-type alkaloid

A novel alkaloid of the meroterpenoid type, oxalicine C **(22),** was extracted from the sea brown alga *Leathesia nana* derived *Penicillium chrysogenum* XNM-12. Compound **(22)** had modest antibacterial activity against the plant pathogen *Ralstonia solanacearum*, with a minimum inhibitory concentration (MIC) value of 8 µg/mL compared to the positive control chloramphenicol with a MIC value of 8 µg/mL [23].

#### Amides and Amines

(±)-Solitumidine E **(23)** and (+)-solitumidine D **(24)** were extracted from the deep-sea-derived *Penicillium solitum* MCCC 3A00215 [14].

### Polyketides

**Fig. 2:**
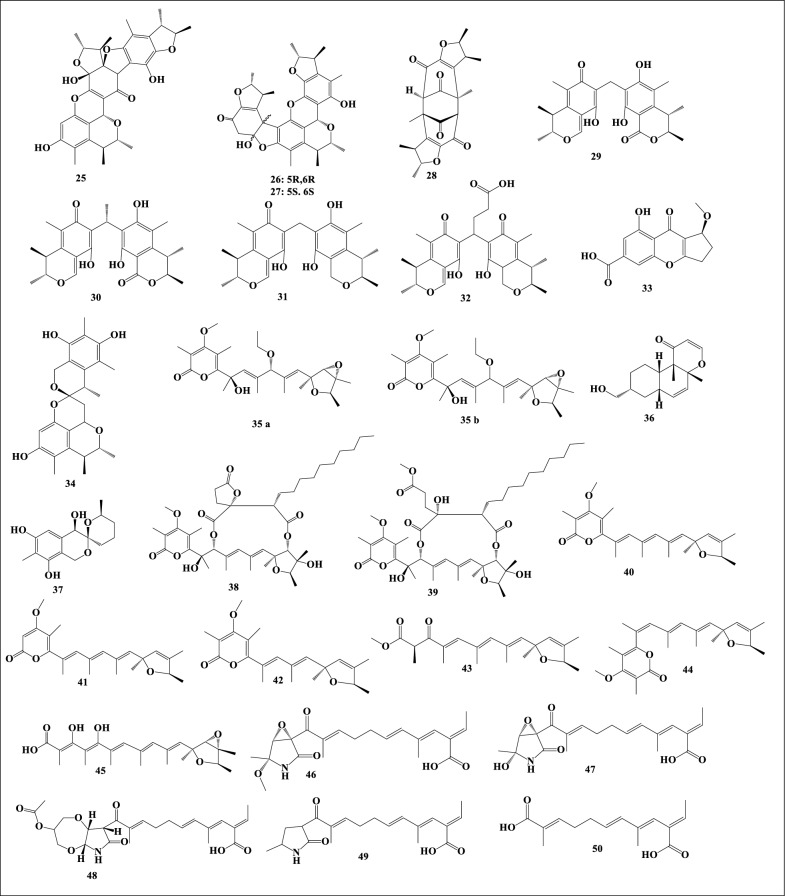
Novel polyketides (citrinins, verrucosidins and fusarins) isolated from *Penicillium* genera associated with marine sources

Ninety-two new polyketides as presented in Fig. 2 of different sub classes were identified from Penicillium genera associated with marine sources.

#### Citrinins

Citrinin is a widely recognized mycotoxin that is frequently present in the byproducts of *Monascus* sp., *Aspergillus* sp. and, *Penicillium* sp.[24]. The production of citrinin in these fungi typically results in the simultaneous presence of various related monomer derivatives, including, 2,3,4-trimethyl-5,7-dihydroxy-2,3-dihydrobenzofuran (TDDF), phenol A acid and decarboxydihydrocitrinin. These derivatives have a tendency to undergo dimerization or trimerization through different pathways, thereby enhancing biological activity, the structural diversity, and complexity of this family of polyketides [25]. Three novel citrinin trimers (neotricitrinols A–C, **25–27**) were produced from the fermentation extract of the deep sea derived *Penicillium citrinum* W23, which is rich in structural diversity. Neotricitrinol A **(25)** has a unique carbon structure consisting of an octacyclic system with a pattern of 5/6/5/5/6/6/6/6. Neotricitrinols B and C **(26–27)** are isomers, with one being endo- and the other exo-, and they have a carbon structure of 5/6/6/6/6/5/6/5. Furthermore, neotricitrinol B **(26)** showed biological effects by inhibiting the formation of fat cells (adipogenic differentiation) and promoting the formation of bone cells (osteoblastogenesis). Besides, it did not show any harmful effects on cells, suggesting its promise in treating osteoporosis. None of the substances exhibited any harmful effects at concentrations up to 10 µM [25]. Also, A novel derivative of citrinin, penicitrinone G **(28),** was obtained from a marine sponge derived *Penicillium citrinum* [26]. Fan et al. clarified in their study that four unique and uncommon carbon-bridged citrinin dimers, specifically dicitrinones G–J (29–32), were extracted from *Penicillium* sp. GGF 16–1-2, which was derived from starfish. Compound **(29)** exhibited notable cytotoxic effects on human pancreatic cancer cell lines BXPC-3 and PANC-1. Compounds **(29–32)** demonstrated potent antifungal effects against *Colletotrichum gloeosporioides*, a significant phytopathogenic fungus that primarily affects tropical fruits and causes severe anthracnose. The LD_50_ values of these compounds ranged from 9.5 to 16.1 µg/mL [27]. The fungus *Penicillium citrinum* SCSIO 41017, which was found in association with the sponge *Callyspongia* sp., yielded two recently discovered polyketides, coniochaetone M **(33)** and xerucitrinin A **(34)** [28].

#### Verrucosidins

A novel set of C-9 epimeric verrucosidin derivatives, named 9-O-ethylpenicyrones A and B **(35a/35b),** were discovered and characterized in the culture extract of *Penicillium cyclopium* SD-413, which originated from sea sediment. These epimers exhibited efficacy against *Aeromonas hydrophila* [29]. Also, it was noted that this sea sediment derived fungus yielded two novel polyketide derivatives, namely 1,2-didehydropeaurantiogriseol E **(36)** and 9-dehydroxysargassopenilline A **(37),** which were successfully isolated and identified. Compound (**36)** showed potent activity against both *V. anguillarum* and *V. harveyi*, with both having a MIC value of 4.0 μg/mL. While Compound **(37)** exhibited strong inhibitory action against *M. luteus*, with a MIC value of 4.0 μg/mL [30]. Moreover, another study revealed that cyclopiumolides A **(38)** and B **(39),** the initial examples of two unique biosynthetically related 13-membered macrolides having a rare verrucosidinol unit combined with a spiculisporic acidic moiety, were discovered in this marine fungus. These compounds **(38)** and **(39)** demonstrated noteworthy cytotoxic effects on the TE-1, SF126, and FaDu tumor cell lines, with IC_50_ values ranging from 5.8 to 17 μM [31]. The deep water derived fungus *Penicillium polonicum* CS-252 was found to have six new derivatives of verrucosidin, which were named poloncosidins A–F **(40–45).** Compound **(40)** exhibited antimicrobial activity against *Vibrio alginolyticus* QDIO-5, and *V. parahemolyticus* QDIO-8, and *Pseudomona aeruginosa* QDIO-4, with MIC values of 8, 4, and 8 µg/mL, respectively. In addition, compound (43) demonstrated inhibition of methicillin-resistant *Staphylococcus aureus* (MRSA) EMBLC-4, with a MIC value of 16 µg/mL. Furthermore, compounds **(43)** and **(45)** had a broad inhibitory effect against *V. alginolyticus* QDIO-5, *V. parahemolyticus* QDIO-8, *P. aeruginosa* QDIO-4, *Klebsiella pneumoniae* EMBLC-3, and, *Escherichia coli* EMBLC-1, with MIC values ranging from 4 to 32 µg/mL [32] (Fig. 2).

#### Fusarins

Song et al. showed in their study that five recently discovered fusarin derivatives, namely steckfusarins A–E **(46–50),** were extracted and characterized from a green algae *Botryocladia* sp. derived *Penicillium steckii* SCSIO 41040. Steckfusarin A **(46)** exhibited limited inhibitory action against lipopolysaccharide-induced nitric oxide (NO) production in RAW 264.7 cells at a dose of 20 µM and demonstrated significant antioxidant activity against DPPH, with an IC_50_ value of 74.5 µg/mL. Also, Compounds **(46–47),** and **(49)** exhibited significant radical scavenging action against DPPH at a concentration of 100 µg/mL with 37.3%, 54.3%, and 45.8% inhibition, respectively [33]. Docking studies revealed that compound **(46)** had the ability to engage with superoxide dismutase at the entry point of the catalytic pocket, with a determined binding affinity of -6.3 kcal/mol and established two hydrogen bonds with residues SER-32 and GLN-153, while forming five hydrophobic contacts with residues LYS-4, VAL-6, HIS-20, ALA-152, and GLN-153. Furthermore, the binding free energy between compound **(47)** and superoxide dismutase was calculated to be -6.6 kcal/mol and six hydrogen bonding interactions between the residues GLN-23, ARG-79, SER-102, LEU-103, and ILE-104 and two hydrophobic contacts with LEU-103 and ALA-105 were observed (Fig. 2).

#### Tanzawaic acids

**Fig. 3:**
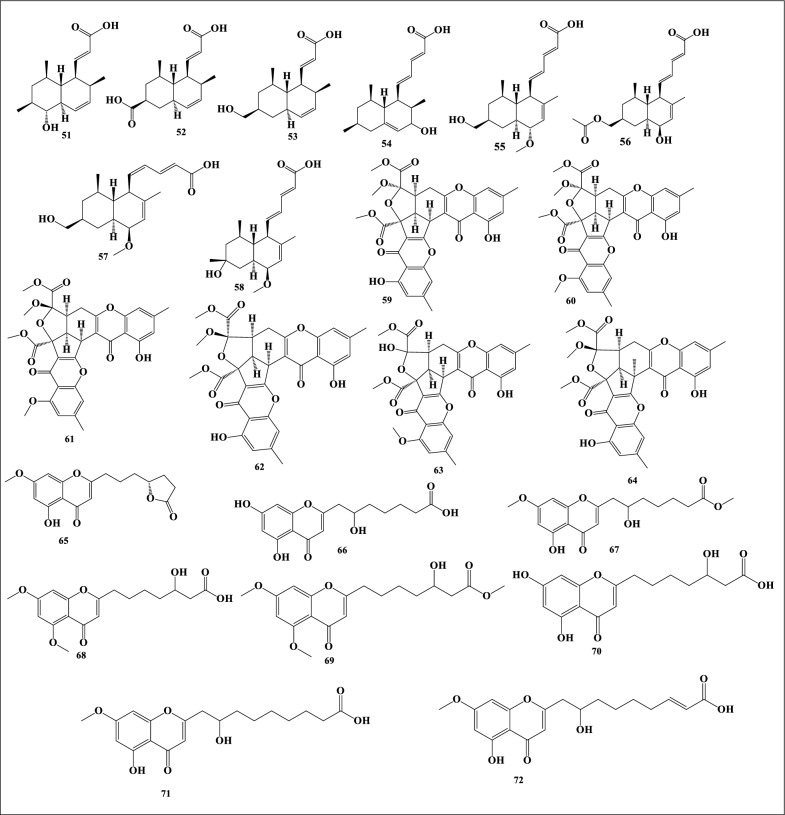
Novel polyketides (tanzawaic acids and chromones) isolated from *Penicillium* genera associated with marine sources

Tanzawaic acids, a type of polyketides, have been found in different strains of *Penicillium* sp. under varied environments [34]. The deep-sea *Acanthogorgiidae* sp. Coral derived *P. steckii* AS-324 yielded four novel steckwaic acids E–H **(51–54)** which are documented for the initial instance, in addition to four fresh analogues **(55–58)** namely 13R-tanzawaic acid S **(55),** 18-O-acetyltanzawaic acid R **(56),** steckwaic acid I **(57),** and10-hydroxytanzawaic acid U **(58).** Among these compounds, compound **(58)** exhibited strong activity against *E. coli*, with a MIC value of 8 µg/mL[35] (Fig. 3).

#### Chromones

Six novel and uncommon chromone derivatives, namely epiremisporine C **(59),** epiremisporine D **(60),** and epiremisporine E **(61),** together with epiremisporine F **(62),** epiremisporine G **(63),** and epiremisporine H **(64),** were extracted from marine-derived *Penicillium citrinum*. Compound **(61)** triggered programmed cell death (apoptosis) through pathways that rely on caspase 3 and suppressed the proliferation of A549 cells. Moreover, compounds **(61)** and **(64)** demonstrated strong cytotoxic effects, with IC_50_ values of and 31.4 ± 3 and 43.8 ± 6.3 µM, respectively, against A549 cells. Besides, these both compounds simultaneously enhanced the expression of bax, a pro-apoptotic protein while inhibiting the expression of Bcl-2, an anti-apoptotic protein. Chu et al. suggested that *Penicillium citrinum* and its specific compounds (particularly **(60- 61)** and **(63—64)** have the potential to be further explored as promising alternatives for preventing or treating several inflammatory illnesses [36, 37]. Another study revealed that eight novel chromone derivatives, designated as penithochromones M − T (**65–72),** were extracted from sea sediment derived *Penicillium thomii*. Compounds **(70–72)** displayed significant inhibition against α-glucosidase, with IC_50_ values ranging from 842 to 1017 µM. These results indicate that these compounds are more potent than the positive control acarbose [38] (Fig. 3).

#### Azaphilones

**Fig. 4:**
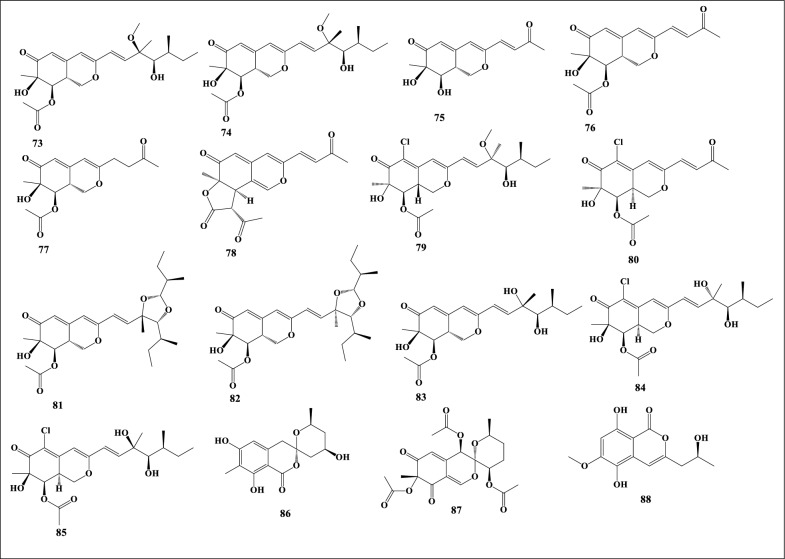
Novel polyketides (azaphilones and isocoumarins) isolated from *Penicillium* genera associated with marine sources

Azaphilones, referred to as fungal pigments, are a type of fungal polyketides that possess an isochroman scaffold consisting of a pyrone-quinone bicyclic core and a quaternary carbon center. Their diverse range of actions, including enzyme inhibitions, antibacterial, cytotoxic, antioxidant, and anti-inflammatory properties, were demonstrated [39]. Eleven novel azaphilones were discovered from the culture of the marine-derived fungus *Penicillium sclerotiorum* E23Y-1A. These include penicilazaphilones I-N **(73–78),** F **(79)** and G **(80),** penidioxolanes C **(81)** and D **(82),** and epi-geumsanol D **(83).** Penidioxolane C **(81)** showed moderate inhibition against various cancer cells, including human liver cancer cells (BEL-7402), human non-small cell lung cancer cells (A549), human myeloid leukemia cells (K562), human hela cervical cancer cells, and human gastric cancer cells (SGC-7901). The IC_50_ values for these cells were 60.6 ± 0.1, 60.1 ± 0.2, 23.9 ± 0.1, 59.3 ± 0.6, and 46.1 ± 0.1 μM, respectively. Furthermore, penicilazaphilone N **(78)** moderately inhibited the production of nitric oxide in LPS-stimulated RAW264.7 cells, with an IC_50_ value of 22.6 ± 2.9 μM. On the other hand, penicilazaphilones F **(79)** and G **(80)** were found to inhibit the production of NO induced by LPS in BV-2 cells, with IC_50_ values of 31.7 ± 1.5 and 34.5 ± 1.4 μM, respectively [40, 41]. Wang et al. conducted study showing that the edible marine macroalgae *Grateloupia* sp. derived *Penicillium sclerotiorum* yielded two novel azaphilones, namely 8a-epi-eupenicilazaphilone C **(84)** and 8a-epi-hypocrellone A **(85).** Compound **(84)** enhanced the SMAD-mediated transcriptional activities that were triggered by TGF-β and Compound **(85)** exhibited specific cytotoxicity against the neuroblastoma cell line SH-SY5Y [42]. Also, the soft coral-derived *Penicillium glabrum* glmu003 yielded two novel azaphilones, namely daldinins G **(86)** and H **(87)** [43] (Fig. 4).

#### Isocoumarins

Ma et al. reported that the marine-derived *Penicillium minioluteum* ZZ1657 yielded isocoumarin, namely peniisocoumarin H **(88)** which showed activity against MRSA, *Candida albicans*, and *Escherichia coli* with a MIC values of 28–33 µg/mL [44] (Fig. 4).

#### Miscellaneous polyketides

**Fig. 5:**
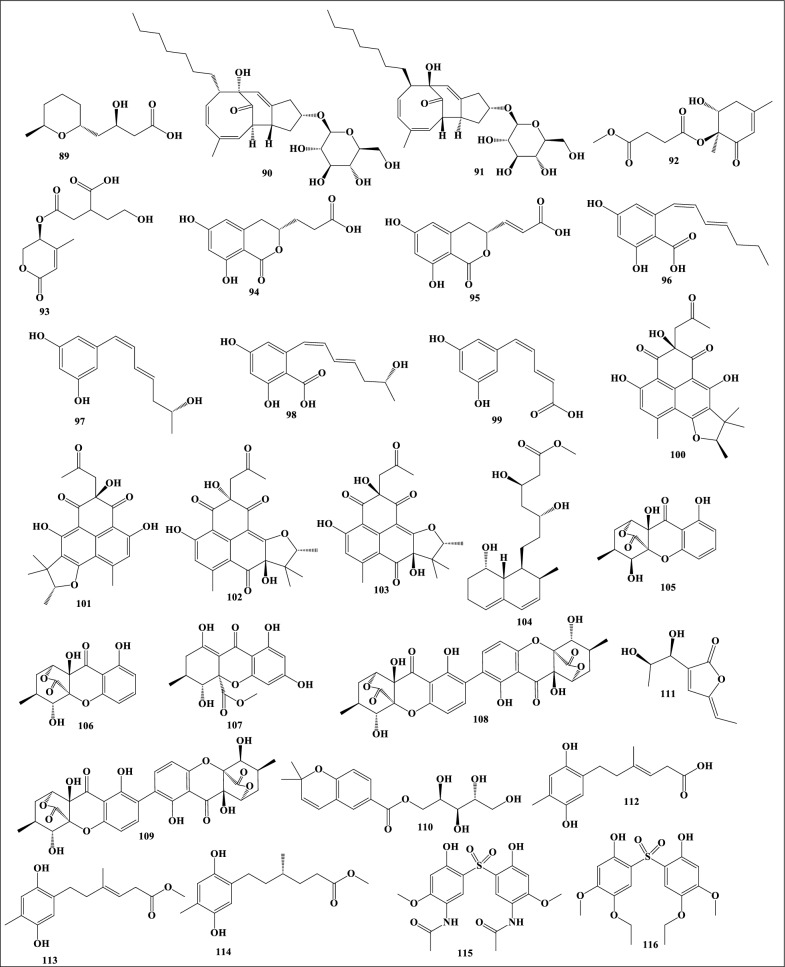
Novel miscellaneous polyketides isolated from *Penicillium* genera associated with marine sources

*Penicillium antarcticum* KMM 4670, a fungus obtained from sea sediment, yielded a new pentaketide derivative called antaketide A **(89)** [45]. Another study by Zhang et al. clarified that an analysis of a *Penicillium copticola* fungus found in a marine sponge revealed the discovery of two novel glycosides, namely 5-glucopenostatin I **(90)** and 5-glycopenostatin F **(91).** These glycosides possess a unique PKS structure with a glucose unit [46]. The marine-derived *Penicillium* sp. TW58-16 yielded eight novel polyketides, namely leptosphaerone D **(92)**,walterolactone E **(93),** 3-[(3S)-3,4-dihydro-6,8-dihydroxy-1- oxo-1H-2-benzopyran-3-yl]-propanoic acid **(94),** (2E)-3-[(3R)-3,4-dihydro-6,8-dihydroxy-1- oxo-1H-2-benzopyran-3-yl]-2-propenoic acid **(95),** 4-carboxy-5-((1Z,3E)-1,3-heptadien1-yl)-1,3-benzenediol **(96),** 5-((R,1Z,3E)-6-hydroxy-1,3-heptadien-1- yl)-1,3-benzenediol **(97),** 4-carboxy-5-((R,1Z,3E)-6-hydroxy-1,3-heptadien-1-yl)- 1,3-benzenediol **(98),** and 5-((1Z,3E)-4-carboxy-1,3-butadienyl-1-yl)-1,3-benzenediol **(99).** Moreover, **(95- 96)** and **(99)** exhibited anti-inflammatory acrivities and compounds **(96–99)**demonstrated potent α-glucosidase inhibitory effects [14, 47]. The marine mangrove-derived *Penicillium herquei* MA-370 was found to contain phenalenone derivatives, including four newly discovered compounds, namely, ( +)-aceatrovenetinone A and B **(100–101),** aceneoherqueinones A and B **(102–103).** Besides, compounds **(102)** and **(103)** exhibited inhibitory activity against angiotensin-I-converting enzyme (ACE), with IC_50_ values of 3.1 and 11.2 μM, respectively [48]. Docking studies revealed that compound **(102)** exhibited the capacity to engage with ACE through the establishment of crucial hydrogen contacts with residues Ala261, Gln618, Trp621, and Asn624. On the other hand, compound **(103)** made significant hydrogen connections with residues Asp358 and Tyr360. Hence, the variance in bioactivities of compounds **(102)** and **(103)** may be attributed to their distinct interactions with various residues of ACE, most likely resulting from the epimerization at C-8. An organized chemical analysis of the fungus *Penicillium solitum* MCCC 3A00215, which was obtained from the deep sea, led to the discovery of a unique polyketide called 15-O-methyl ML-236A **(104)** [49]. Yong et al. reported in their study that the marine-derived *Penicillium* sp. ZZ1750 yielded five polyhydroxanthones, namely ergochromes C-G **(105–109).** These compounds **(105–109),** had modest antibacterial effects against C. albicans and MRSA, with MIC values ranging from 16 to 36 µg/mL [50]. The marine-derived *Penicillium* sp. MCCC 3A00228 yielded a novel benzopyran derivative, D-arabinitol-anofinicate **(110),** which had a mild stimulatory effect on the transcription of the orphan nuclear receptor Nur77 [51]. The marine red alga *Grateloupia turuturu*-associated *Penicillium chrysogenum* LD-201810 yielded a novel pentaketide derivative, penilactonol A **(111)** [52]. The fungus *Penicillium* sp. HDN151272, isolated from an *Antarctica* sponge, produced three novel hydroquinone derivatives named ketidocillinones A–C **(112–114)**. Ketidocillinones B and C **(113 and 114)** demonstrated strong antibacterial efficacy against MRCNS (methicillin-resistant coagulase-negative *staphylococci*), *Mycobacterium phlei*, and *Pseudomonas aeurigenosa* [53]. The marine red alga *Laurencia obtusa*-associated *Penicillium aculeatum* yielded two novel sulfonyl metabolites, namely pensulfonamide **(115)** pensulfonoxy **(116)**. Compound **(115)** displayed fungicidal properties against *Candida albicans*. Moreover, this compound **(115)** exhibited the highest level of selective toxicity towards MCF-7 cells, whereas compound **(116)** showed only moderate action against HCT-116 cells [54] (Fig. 5).

### Terpenoids

Seventy terpenoids belonging to different sub classes were identified from Penicillium genera that are associated with marine sources; they are as follows:

#### Sesquiterpenes

**Fig. 6:**
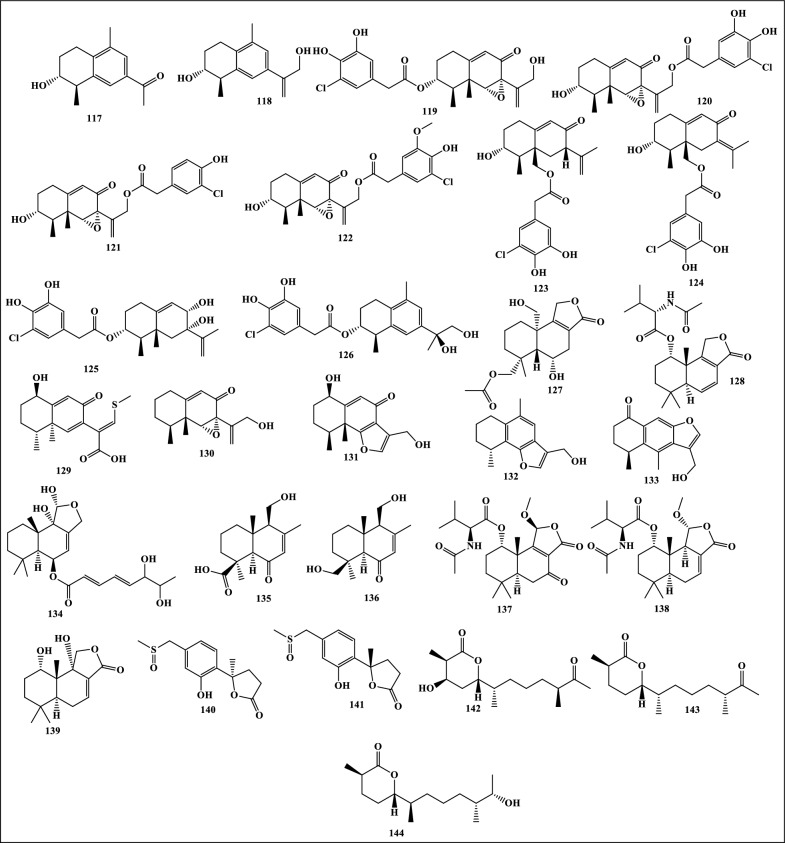
*Sesquiterpenes* isolated from *Penicillium* genera associated with marine sources

Eremophilanes are a group of sesquiterpenes distinguished by the presence of a bicyclic structure. Marine-derived Penicillium fungi were found to produce a collection of eremophilanes with unique structures and notable bioactivities. From the fungus *Penicillium copticola* generated from sponges, ten eremophilanes that were previously characterized were isolated. These eremophilanes are named copteremophilanes A-J **(117–126).** The bioassay results demonstrated that the isolated eremophilanes had inhibitory effects on tumour cell lines, which were attributed to structural variations. Compounds **(120–121)** exclusively reduced the growth of HCT-8 cells, while compound **(124)** specifically suppressed the growth of A549 human non-small cell lung cancer cells. Furthermore, the noncytotoxic compounds, including **(123),** exhibited a neuroprotective impact. In this regard, eremophilanes possess the potential to be developed as drugs with anticancer or neuroprotective properties following structural alteration [46]. The rhizosphere soil of mangrove plant *Avicennia marina* fungus *Penicillium* sp. N-5 yielded a novel drimane sesquiterpenoid, astellolide Q **(127)** [55]. Another study proved that the marine-derived *Penicillium* strain ZZ1283 yielded a novel compound belonging to the drimane-type sesquiterpene lactones conjugated with N-acetyl-L-valine, namely purpuride D **(128)** which exhibited antibacterial properties, with MIC values of 8 μg/mL against *C. albicans*, 3 μg/mL against E. coli, and 4 μg/mL against MRSA [56]. The mangrove-derived *Penicillium* sp. HDN13-494 yielded five recently discovered sesquiterpenoids, namely phomenone A–B **(129–130)** and citreobenzofuran D–F **(131–133).** Phenomenon B **(130)** had a moderate level of activity against *Bacillus subtilis*, with a MIC value of 6.2 µM [57]. Huang et al. conducted a study showing that the marine –derived *Penicillium chrysogenum* LD-201810 yielded a novel compound classified as a drimane sesquiterpene ester, namely Chrysoride A **(134)** which shown moderate cytotoxicity against HeLa and HepG2 cancer cell lines, with IC_50_ values of 35.6 and 28.9 μM, respectively [58]. Gou et al. clarified in their study that the ethyl acetate (EtOAc) extract of the fungus *Penicillium* sp. TW58-16 yielded two novel drimane sesquiterpenes, namely (4S,5R,9S,10R)-11-hydroxy-13-carboxy-drim-7-en-6-one **(135)** and (4S,5R,9S,10R)-11,13-dihydroxy-drim-7-en-6-one **(136).** Compound **(136)** shown significant inhibition against α-glucosidase, with 35.4% rate [47]. Ma et al. reported that the marine-sourced *Penicillium minioluteum* ZZ1657 yielded three drimane sesquiterpenoids, namely purpurides E–G **(137–139).** It was noted that purpuride G **(139)** shown strong antiproliferative properties against human glioma U87MG and U251 cells. Moreover, purpurides E and F **(137–138)** exhibited significant antimicrobial properties by suppressing *C. albicans*, *E. coli*, and MRSA growth [44]. The marine algal-derived endophytic fungus *Penicillium chrysogenum* LD-201810 yielded a pair of novel enantiomers, namely ( ±)-1-methylsulfinyl-1-hydroxyboivinianin A **(140–141),** which are derivatives of nor-bisabolane [59]. The marine red algal-derived *Penicillium chermesinum* EN-480 yielded Three novel compounds, namely chermesiterpenoids A–C **(142–144).** Compounds **(143)** and **(144)** shown significant antimicrobial activity against plant pathogenic fungus *Colletottichum gloeosporioides*, human pathogen *Escherichia coli*, and aquatic pathogenic bacteria *Micrococcus luteus*, *Vibrio parahaemolyticus*, and *V. anguillarum* [60] (Fig. 6).

#### Diterpenes

**Fig. 7:**
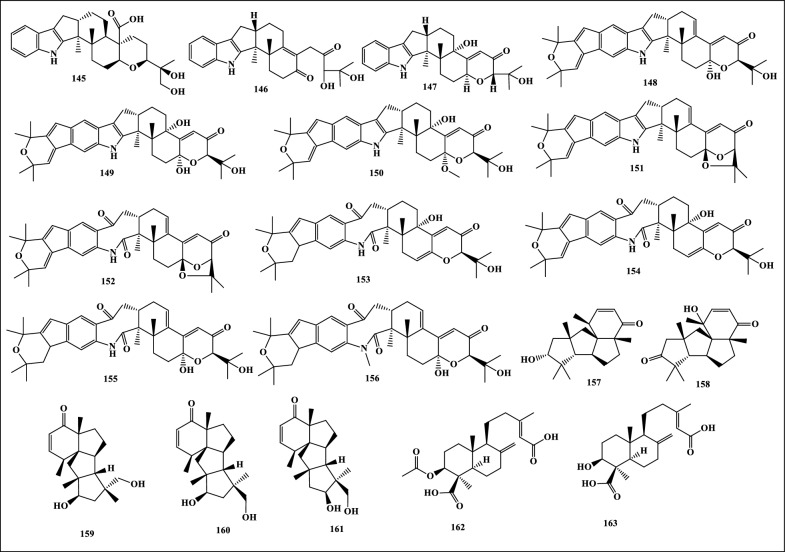
Diterpenes isolated from *Penicillium* genera associated with marine sources

Indole terpenoids are a significant group of natural compounds that exhibit a wide range of biological functions and have many structural configurations. The marine-derived fungus *Penicillium citrinum* ZSS-9 yielded a novel indole-diterpenoid compound, named penijanthine E **(145),** which was isolated from the PDB culture. Compound **(145)** exhibited antiviral efficacy against influenza A virus (IAV) strains A/PR/8/34 (H1N1) and A/WSN/33(H1N1) [61].The marine-derived fungus *Penicillium* sp. KFD28 yielded two indole-diterpenoid compounds, namely penerpene J **(146)** and epipaxilline **(147).** Compound **(146)** shown inhibitory activity against TCPTP, with an IC_50_ value of 14.7 μM. Moreover, compound **(146)** and compound **(147)** exhibited inhibitory effects on PTP1B, with IC_50_ values of 9.5 and 31.5 μM, respectively [62]. Co-culturing fungi is becoming recognized as a valuable method for investigating the wide range of secondary metabolites produced by fungi [63]. Evidence demonstrated that fungal co-culture has the ability to stimulate dormant genes in fungi, leading to the identification of previously unknown secondary metabolites through novel biosynthetic pathways [64]. Moreover, the secondary metabolites acquired by the co-culture process are linked to the fungi's defense systems, which frequently exhibit notable biological properties. It was reported that nine novel indole-diterpenes, named janthinellumines A–I **(148–156),** were isolated by co-culturing the marine-derived fungus *Penicillium janthinellium* with *Paecilomyces formosus*. Compounds, **(148,149** and **154)** exhibited noteworthy activity against two strains (A/WSN/33 (H1N1) and A/Hong Kong/1/68 (H3N2)) with CC_50_ values of 70.7, 132.4, and 134.7 μg/mL, respectively, showing their comparatively low toxicity. Besides, Compound **(148)** and compound **(155)** had limited effectiveness against *V. anguillarum*, with MIC values of 12.5 and 25.0 μg/mL, respectively [65]. The molecular binding site between NA protein and compounds (**148,149** and **154**) was identified by biomolecular docking using AutodockTools. In the binding pocket, compounds (**148,149** and **154**) have the ability to establish several hydrogen bonds with the amine of the Arg371 side chain. In this regard, the side chain of Arg371 has the ability to interact with compounds (**148, 149** and **154**). This interaction is one of the factors contributing to the anti-influenza A virus activities of these compounds. The marine sediment-derived *Penicillium antarcticum* KMM 4670 yielded two novel cyclopiane diterpenes, namely 13-epi-conidiogenone F **(157)** and 4-hydroxyleptosphin C **(158).** Compound **(157)** has been discovered to possess inhibitory properties against sortase A and shows a potential as an effective drug against staphylococcal infections [45]. Another study by Li et al. reported that the marine sediment-derived *Penicillium* sp. TJ403-2 yielded three novel and uncommon cyclopiane diterpenes, namely 12β-Hydroxy conidiogenone D **(159),** 12β-Hydroxy conidiogenone C **(160),** and 13*β*-hydroxy conidiogenone C **(161).** Compound **(161)** significantly reduced the production of NO caused by LPS, with an IC_50_ value of 2.2 ± 0.2 μM. This value was three times lower than that of indomethacin. In this regard, compound **(161)** demonstrated potential as an anti-inflammatory drug by effectively inhibiting inflammatory mediators and cytokines in laboratory tests [66]. Labdane-type diterpenoids are a highly prevalent class of secondary metabolites found in plants, fungi, and marine organisms. Cheng et al. conducted a study showing that The EtOAc extract of the deep-sea derived strain *Penicillium thomii* YPGA3 yielded two newly discovered labdane-type diterpenoids, namely 3*β*-Acetoxy-agathic acid **(162)** and 3*β*-hydroxy-agathic acid **(163)**[67] (Fig. 7).

##### Sesterterpenoid

**Fig. 8:**
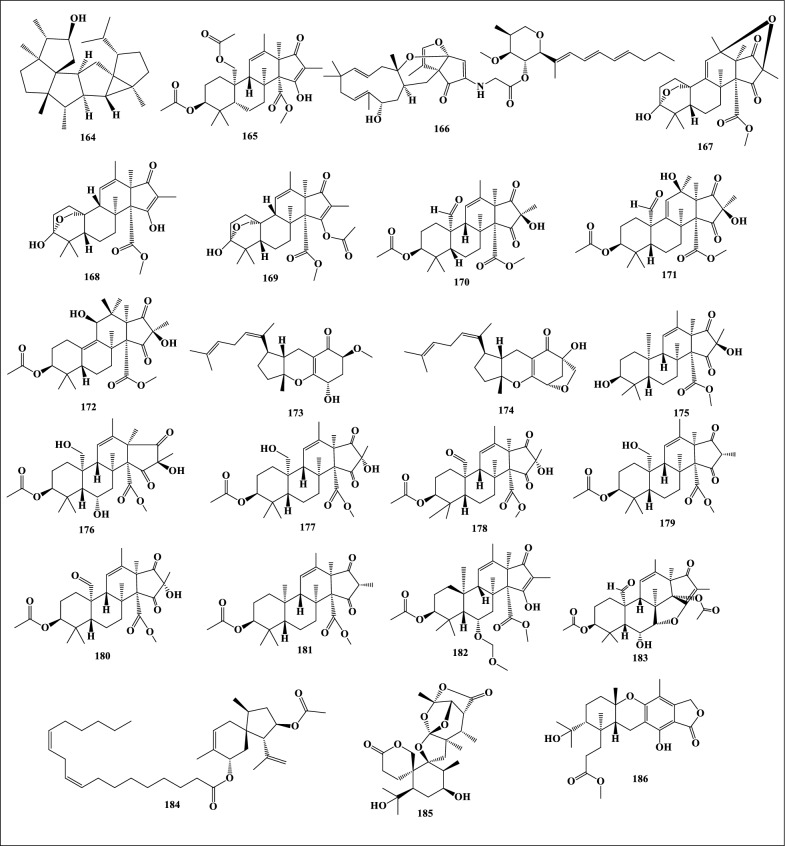
Sesterterpenoid and Meroterpenes isolated from *Penicillium* genera associated with marine sources

The methanol extract of the culture broth of *Penicillium oxalicum* M893 yielded a novel sesterterpenoid, oxaliterpenoid **(164)** which exhibited strong antibacterial effects against Gram-positive bacteria *B. cereus* (ATCC14579), *E. faecalis* (ATCC299212), and *S. aureus* (ATCC25923),as well as the yeast *Candida albicans* (ATCC10231), with MIC values ranging from 32 to 128 µg/mL [68] (Fig. 8).

##### Meroterpenes

The term "meroterpenoid" refers to a category of secondary metabolites that are partially produced from the terpenoid biosynthetic pathway. Meroterpenoids have extensive structural variability, ranging from simple molecules composed of a prenyl unit linked to a phenolic derivative, to more intricate meroterpenoids featuring functionalized carbon chains [69]. A novel meroterpenoid derivative, known as andrastin I **(165),** was extracted from a fungus called *Penicillium ochrochloron*, which was obtained from a marine source [70]. Another study by Yong et al. clarified that the marine fungus *Penicillium* sp. ZZ1750 yielded Penipyridinone B **(166)** which shown inhibitory effects on the growth of glioma cells [18]. Andrastin-type meroterpenoids are distinguished by a 6,6,6,5-tetra-carbocyclic structure that originates from a combination of a sesquiterpene and a tetraketide. These compounds are mostly discovered in the fungus genus *Penicillium*. The rhizosphere soil cultures of the mangrove plant *Avicennia marina*, namely from the fungus *Penicillium* sp. yielded three novel meroterpenoids of the andrastin-type, namely hemiacetalmeroterpenoids A–C **(167–169).** Compound **(167)** demonstrated significant antimicrobial effects against *Colletrichum gloeosporioides* and *Penicillium italicum*, with MIC values of 6.2 µg/mL and exhibited inhibitory effects on *Bacillus subtilis* at a dosage of 6.2 µg/mL [55]. Ren et al. reported in their study that the culture extract of a marine-derived *Penicillium* sp. yielded three novel andrastin-type meroterpenoids, namely penimeroterpenoids A–C **(170–172).** Moreover, compound **(170)** exhibited mild cytotoxic effects on SW480, HCT116, and A549 cell lines [71]. *Crella* sponge-derived *Penicillium* sp. NBUF154 yielded two novel meroterpenoids, Guignardones Y–Z **(173–174).** Compound **(173)** exhibited strong inhibitory effects against human enterovirus 71 (EV71)[72]. By changing the culture media from rice to oat, the OSMAC strategy was used to produce and isolate nine new andrastone meroterpenoids **(175–183)** from the deep-sea derived *Penicillium allii-sativi* MCCC 3A00580. These meroterpenoids include andrastone B **(175),** andrastone C **(176),** andrastone D **(177),** andrastone E **(178),** andrastone F **(179),** andrastone G **(180),** andrastone H **(181),** citrehybridonol B **(182),** and andrastin G **(183).** Andrastone B **(175)** caused a considerable reduction in degranulation, with IC_50_ values of 40.4 μM. It has the potential to reduce the release of histamine and the synthesis of TNF-α in a dose-dependent manner. Furthermore, it inhibited the build-up of Ca2 + in RBL-2H3 cells [73]. Andrastone C **(176)** was also isolated from the Mariana Trench Sediment-Derived *Penicillium* sp. SY2107 and shown antibacterial properties against methicillin-resistant *Staphylococcus aureus* (MRSA), *Escherichia coli*, and *Candida albicans* [74]. The co-culture broth of two marine-derived fungus, *P. bilaiae* MA-267 and *P. chermesinum* EN-480 yielded two novel meroterpenoid derivatives, identified as chermebilaenes A **(184)** and B **(185).** Compound **(184)** exhibited strong inhibitory effects against *Ceratobasidium cornigerum* and *Edwardsiella tarda* [75]. The ethyl acetate extract of the deep-sea derived strain *Penicillium thomii* YPGA3 yielded a novel Austalide Y meroterpenoid **(186)** which exhibited a low level of inhibition against MDA-MB-468 cells, with an IC_50_ value of 38.9 μM [67] (Fig. 8).

##### Miscellaneous terpenoids

**Fig. 9:**
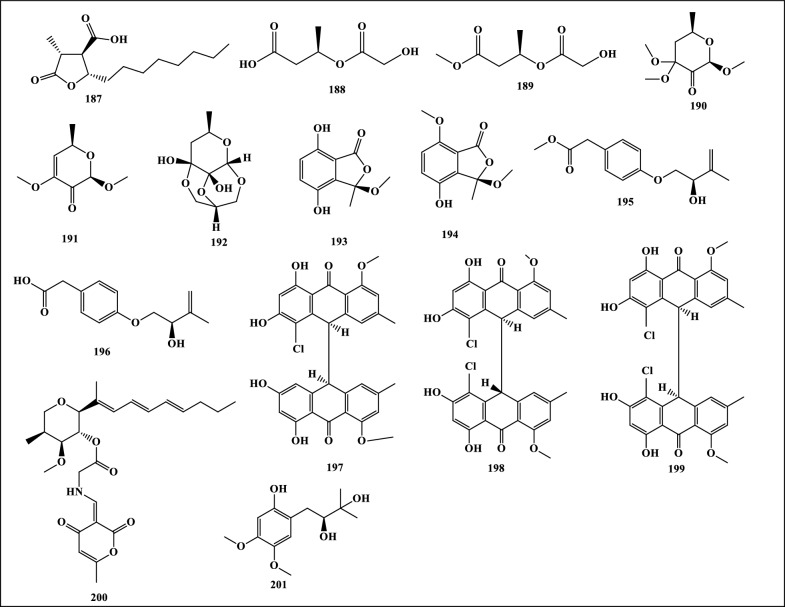
Miscellaneous terpenoids isolated from *Penicillium* genera associated with marine sources

Fifteen additional miscellaneous terpenoids 4 were identified from Penicillium genera associated with marine sources and are represented in Fig. 9. The co-cultivation of the fungus *Penicillium ochrochloron* with *Bacillus subtilis* resulted in the production of a new natural compound called ochrochloronic acid **(187),** which belongs to the butyrolactone family. The previously unreported derivatives of (R)-3-hydroxybutyric acid and glycolic acid, namely penisterines A-E **(188–192),** were discovered and identified for the first time in a marine brown alga *Sargassum cristaefolium*-derived fungal strain, *Penicillium sumatraense* SC29. Penisterine D **(191)** exhibits anti-angiogenic effects in both human endothelial progenitor cells (EPCs) and a transgenic zebrafish model. In this regard, it is a promising option for more preclinical research [76]. The culture filtrate of the endophytic *Penicillium chrysogenum* LD-201810, which is generated from maritime algae, yielded two novel phthalides, namely Chrysoalide A **(193)** and Chrysoalide B **(194)** [59]. The solid culture of *Penicillium chrysogenum* LD-201810, which was obtained from the marine red alga *Grateloupia turuturu* yielded two novel hydroxyphenylacetic acid derivatives, namely (2’R)-westerdijkin A **(195)** and (2’R)-stachyline B **(196).** Compound **(195)** demonstrated cytotoxic effects on the HepG2 cell line, with an IC_50_ value of 22 µM [52]. Morehouse et al. reported in their study that from the marine alga *Petalonia fascia*-derived *Penicillium roseopurpureum* (KP1-135C) yielded three halogenated bianthrones, namely neobulgarone D, neobulgarone E, and neobulgarone F **(197–199).** These compounds exhibited specific antibacterial effects against *Mycobacterium* TB H37Ra and *Staphylococcus aureus* [77]. The EtOAc extract of the marine-derived fungus *Penicillium janthinellum*, which was acquired from a sediment sample, yielded a novel derivative called restricticin B **(200)** which shown anti-neuroinflammatory properties by inhibiting the synthesis of pro-inflammatory substances in activated microglial cells [78]. The culture of the fungus *Penicillium chrysogenum* ZZ1151, which was collected from Indonesian mangrove sediment, yielded a novel tetrasubstituted benzene derivative, peniprenylphenol A **(201)** which exhibited potent antimicrobial effects against *Candida albicans*, *Escherichia coli*, and MRSA, with MIC values 13, 13, and 6 µg/mL, respectively [79].

### Fatty acids

**Fig. 10:**
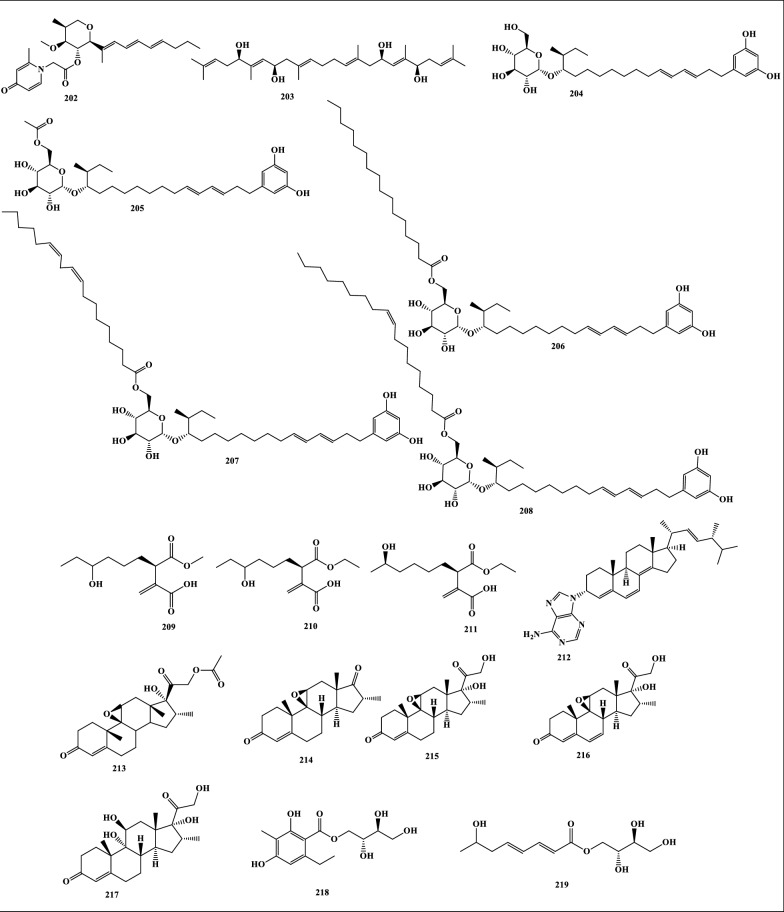
Fatty acids, steroids and polysaccharides isolated from *Penicillium* genera associated with marine sources

Ten fatty acids were identified from Penicillium genera associated with marine sources and presented in Fig. 10. Yong et al. conducted a study showing that the marine-associated *Penicillium* sp. ZZ1750 in a rice medium yielded seven newly identified chemicals, specifically penipyridinone A (202), penidifarnesylin A **(203),** and peniresorcinosides A–E **(204–208).** Penidifarnesylin A **(203)** exhibited antiproliferative action with IC_50_ values of 27.6 µM against U251 cells and 5.9 µM against U87MG cells. Furthermore, peniresorcinosides C–E **(206–208)** displayed moderate antiglioma action against U87MG cells. While, Peniresorcinosides A and B **(204–205)** shown significant antiproliferative effects on both glioma U251 cells and U87MG [80]. *Penicillium antarcticum*, a marine-derived fungus, yielded methyl 8-hydroxyhexylitaconate **(209),** ethyl 8-hydroxyhexylitaconate **(210),** and ethyl 9-hydroxyhexylitaconate **(211)** [81].

### Steroids

Six steroids were identified from Penicillium genera associated with marine sources and presented in Fig. 10. Hou et al. clarified in their study that the EtOAc extract of mangrove sediment-derived *Penicillium brefeldianum* ABC190807 yielded a novel purinyl-steroid, specifically ergosta-4,6,8(14),22-tetraen-3-(6-amino-9H-purin-9-yl) **(212)**[82]. The sponge species *Callyspongia* sp-derived *Penicillium citrinum* SCSIO 41017 yielded a novel steroid, specifically 16a-methylpregna-17a,19-dihydroxy-(9,11)-epoxy-4-ene-3,18-dione-20-acetoxy **(213)** which exhibited a moderate level of activity against (A549, MCF-7, SF-268 and HepG-2) cell lines, with IC_50_ values ranging from 13.5 to 18.0 μM [28]. The soft coral-derived *Penicillium* sp. SCSIO41201 yielded four novel steroid derivatives, namely Penicildiones A-D **(214–217)** [83].

### Polysaccharides

Two polysaccharides of different sub classes were identified from Penicillium genera associated with marine sources and presented in Fig. 10. *Penicillium chrysogenum* XNM-12, an endophytic fungus obtained from the coastal brown alga *Leathesia nana*, yielded two novel erythritol derivatives, namely penicierythritols A and B **(218–119).** Compound **(218)** demonstrated a modest level of antifungal activity against the plant pathogen *A. alternata*, with a MIC value of 8 µg/mL and antibacterial activity against the plant pathogen *Ralstonia solanacearum*, with a MIC value of 4 µg/mL compared to the positive control chloramphenicol with a MIC value of 8 µg/mL [23].

## The biological activities of secondary metabolites from marine-derived endosymbiotic Penicillium fungi

Many biological activities were assigned to the secondary metabolites from marine-derived endosymbiotic *Penicillium* fungi such as antibacterial, cytotoxic, anti-inflammatory, antiviral, antifungal, neuroprotective, and anti-glioma in addition to many other activities. Different biological activities were illustrated in Table 1. Meanwhile, Table 2 demonstrates the docking results of isolated novel and established compounds from marine-derived *Penicillium* fungi against their target enzymes including sortase A, PARP1, SARS-CoV-2 Mpro, PI3K, peptide deformylases, iNOS, ACE, SHP2, monoamine oxidases, PTP1B, TCPTP, α-glucosidase and VEGFR2, the activities supporting docking, the binding energies, bonding interactions, and related references.

**Table 1:** The biological activities of secondary metabolites from marine-derived endosymbiotic Penicillium fungi

Year	Metabolites	Producing Strain	Environment Source	Activity	Refs.
2025	Shearinine R	*Penicillium* sp. N4-3	Marine organism	Antibacterial and cytotoxic	[84]
2025	Penibinaphthol C	*Penicillium* sp. HQ1-23	Marine organism	Anti-neuroinflammatory	[85]
2025	Penicacids L − N	*Penicillium* sp. HN-66	Marine sediment	Antimicrobial	[86]
2025	PCO-1	*Penicillium citrinum* SCAU-268	Marine organism	Immunomodulatory	[87]
2024	Dicitrinone F, stoloniferol B, (2S)-2,3- dihydro-7-hydroxy-6,8-dimethyl-2[(E)-prop-1-enyl]-chroman-4-one, dihydrocitrinin, phenol acid, phenol A, 3β-hydroxy-5,9-epoxy-(22E,24R)-ergosta-7,22-dien-6-one, melithasterol B	*Penicillium Citrinum* VM6	Marine organism	Antimicrobial and cytotoxic	[88]
2024	Penipiperazine A, Penipiperazine B	*Penicillium brasilianum*	Marine organism	Anti-inflammatory	[13]
2024	Penifuranone A	*Penicillium crustosum* SCNU-F0006	Mangrove	Anti-inflammatory, antioxidant and antimicrobial	[89]
2023	Steckfusarins A–E	*Penicillium steckii* SCSIO41040	Marine organism	Antioxidant and anti-inflammatory	[33]
2023	Neotricitrinols A–C	*Penicillium citrinum* W23	Deep-sea	Antiosteoporosis	[25]
2023	Oxaliterpenoid	*Penicillium oxalicum* M893	Marine organism	Antimicrobial	[68]
2023	Cerevisterol, ergosterol peroxide, and (3b,5a,22E)-ergosta-6,8(14),22-triene-3,5-diol	*Penicillium levitum* N33.2	Marine organism	Cytotoxic and inhibitory activity against alpha-glucosidase	[90]
2023	Penicilazaphilones I–N, epi-geumsanol D, penidioxolanes C and D	*Penicillium sclerotiorum* E23Y-1A	Marine sponge *Holoxea* sp.	Anti-inflammatory and cytotoxic	[41]
2023	Toluquinol	*Penicillium griseofulvum*	Marine algae	Antimicrobial	[91]
2023	Andrastin I ochrochloronic acid (from Co-cultivation)	*Penicillium ochrochloron*	Marine organism	_	[70]
2023	9-O-ethylpenicyrones A and B (3a/3b)	*Penicillium cyclopium* SD-413	Marine sediment	Antibacterial	[29]
2023	Averufin, aspergilol-A, sulochrin, monomethyl sulochrin, methyl emodin, citreorosein, and diorcinol	*Penicillium verruculosum*	Sponge (*Spongia officinalis*)	Anticancer	[92]
2023	penijanthine E	*Penicillium citrinum* ZSS-9	Marine organism	Antiviral	[61]
2023	4-Hydroxyleptosphin C, 13-epi-conidiogenone F and antaketide A	*Penicillium antarcticum* KMM 4670	Marine sediment	Anti-*staphylococcal* agent	[45]
2023	Perpyrrospirone A	*Penicillium citrinum*	Marine organism	Cytotoxic	[15]
2023	Janthinellumines A–I	*Penicillium janthinellium* Co-culturing with *Paecilomyces formosus*	Marine organism	Anti-influenza A	[65]
2023	Penicinolone	*Penicillium *sp. SCSIO41033	Sponge	–	[93]
2022	Pyrrospirones K-Q	*Penicillium* sp. SCSIO 41512	Marine organism	Antibacterial	[16]
2022	Penipyridinone B, 11S-( −)-penilloid A and 11R,14E-( +)-penilloid A	*Penicillium* sp. ZZ1750	Marine organism	Antiglioma	[18]
2022	Poloncosidins A–F	*Penicillium polonicum* CS-252	Deep-sea	Antibacterial	[32]
2022	Steckwaic acids E–H, 10-Hydroxytanzawaic acid U, 18-O-Acetyltanzawaic acid R, Steckwaic acid I, 13R-Tanzawaic acid S	*Penicillium steckii* AS-324	Deep-sea *Acanthogorgiidae* sp. coral	Antibacterial	[35]
2022	Dicitrinones G–J	*Penicillium* sp. GGF16-1–2	Starfish	Antifungal and cytotoxic	[27]
2022	Cyclopiumolides A and B	*Penicillium cyclopium* SD-413	Deep sea	Cytotoxic	[31]
2022	Copteremophilanes A–J, 5-glycopenostatin F and 5-glucopenostatin I	*Penicillium Copticola*	Marine organism	Neuroprotective and cytotoxic	[46]
2022	(8S,9R,12R,18S)-12-Hydroxy-fumitremorgin B, walterolactone E and leptosphaerone D	*Penicillium* sp. TW58-16	Marine organism	Antibacterial	[14]
2022	Penicilazaphilones F and G	*Penicillium sclerotiorum* E23Y-1A	Marine organism	Inhibited the lipopolysaccharide-induced production of nitric oxide	[40]
2022	Penisterines A and C–E	*Penicillium sumatraense* SC29	Marine brown alga *Sargassum cristaefolium*	Anti-angiogenic	[76]
2022	Hemiacetalmeroterpenoids A–C and astellolide Q	*Penicillium* sp. N-5	Rhizosphere soil of mangrove plant *Avicennia* marina	Antimicrobial	[55]
2022	Citrinadin C	*Penicillium citrinum*	Deep-sea sediment	Cytotoxic	[20]
2022	Pensulfonoxy and pensulfonamide	*Penicillium aculeatum*	Marine red alga *Laurencia obtusa*	Antifungal and cytotoxic	[54]
2022	Citreobenzofuran D–F and phomenone A–B	*Penicillium* sp. HDN13-494	Mangrove	Antibacterial	[57]
2022	Guignardones Y–Z	*Penicillium* sp. NBUF154	deep *Crella* sponge	antiviral	[72]
2022	Chrysoride A	*Penicillium chrysogenum* LD-201810	Marine organism	Cytotoxic	[58]
2022	Peniprenylphenol A	*Penicillium chrysogenum* ZZ1151	Mangrove sediment	–	V
2022	Isocoumarins penicillol A and penicillol B, citreoviridin H and citreoviridin I	*Penicillium* sp. BJR-P2	Mangrove	Anti-inflammatory	[94]
2021	Epiremisporine C, epiremisporine D, and epiremisporine E	*Penicillium citrinum*	Marine organism	Anti-inflammatory and cytotoxic	[36]
2021	Epiremisporine F, epiremisporine G and epiremisporine H	*Penicillium citrinum*	Marine organism	Anti-inflammatory and cytotoxic	[37]
2021	Talumarin A, aspergillumarin A, andrastin A, Clavatol, 3-acetylphenol, methyl 2,5-dihydro-4-hydroxy-5-oxo-3-phenyl-2-furanpropanoate, Emodin and 2-chloroemodin	*Penicillium ochrochloron*	Marine organism	_	[95]
2021	Penimeroterpenoids A–C	*Penicillium* sp	Marine organism	Cytotoxic	[71]
2021	Purpuride D	*Penicillium* strain ZZ1283	Marine organism	Antimicrobial	[56]
2021	Penerpenes K-N	*Penicillium* sp. KFD28	Marine	Cytotoxic	[96]
2021	Peniresorcinosides A–E, penidifarnesylin A, and penipyridinone A	*Penicillium* sp. ZZ1750	Marine organism	Antiproliferative	[80]
2021	Ergochrome C and ergochromes D–G	*Penicillium* sp. ZZ1750	Marine organism	Antimicrobial	[50]
2021	D-Arabinitol-anofinicate	*Penicillium* sp. MCCC 3A00228	Marine organism	Exhibited a weak activation effect on orphan nuclear receptor Nur77 transcription	[51]
2021	( +) − 1 Methylsulfinyl-1hydroxyboivinianin A, (−) − 1 methylsulfinyl-1-hydroxyboivinianin A, Chrysoalide A and Chrysoalide B	*Penicillium chrysogenum* LD-201810	Marine algae	Hydroxysydonic acid showed strong inhibition against *B. cinerea*	[59]
2021	(4S,5R,9S,10R)-11,13-Dihydroxy-drim-7-en-6-one, (4S,5R,9S,10R)-11-hydroxy-13-carboxy-drim-7-en-6-one, 5-((R,1Z,3E)-6-hydroxy-1,3-heptadien-1-yl)-1,3-benzenediol, 4-carboxy-5-((R,1Z,3E)-6-hydroxy-1,3-heptadien-1-yl)- 1,3-benzenediol, 4-carboxy-5-((1Z,3E)-1,3-heptadien1-yl)-1,3-benzenediol, 5-((1Z,3E)-4-carboxy-1,3-butadienyl-1-yl)-1,3-benzenediol, (2E)-3-[(3R)-3,4-dihydro-6,8-dihydroxy-1- oxo-1H-2-benzopyran-3-yl]-2-propenoic acid and 3-[(3S)-3,4-dihydro-6,8-dihydroxy-1- oxo-1H-2-benzopyran-3-yl]-propanoic acid	*Penicillium* sp. TW58-16	Marine organism	Anti-inflammatory and α-glucosidase inhibitory effects	[47]
2021	Citrehybridonol B, Andrastin G, Andrastone B, Andrastone C, Andrastone D, Andrastone E, Andrastone F, Andrastone G and Andrastone H	*Penicillium allii-sativi* MCCC 3A00580	Deep-sea	Anti-allergic	[73]
2021	Aceneoherqueinones A and B, ( +)-aceatrovenetinone A and ( +)-aceatrovenetinone B	*Penicillium herquei* MA-37	Marine mangrove	Aceneoherqueinones A and B displayed inhibitory activity against angiotensin-I-converting enzyme (ACE)	[48]
2021	(Ergosta-4,6,8(14),22-tetraen-3-(6-amino-9H-purin-9-yl)	*Penicillium brefeldianum* ABC19080	Mangrove sediments	Larvicidal	[82]
2021	Epipaxilline and penerpene J	*Penicillium* sp. KFD28	Marine organism	Inhibitory activities against PTP1B	[62]
2021	8a-epi-Hypocrellone A and 8a-epi-eupenicilazaphilone C	*Penicillium sclerotiorum*	Marine *macroalgae Grateloupia* sp	Anti-inflammatory	[42]
2021	15-*O*-Methyl ML-236A, ( +)-solitumidine D and ( ±)-solitumidine E	*Penicillium solitum* MCCC 3A00215	Deep-sea	Cytotoxic	[49]
2021	Penithochromones M − T	*Penicillium thomii*	Marine organism	α-Glucosidase inhibitory activity	[38]
2021	Xerucitrinin A, coniochaetone M, 16a-methylpregna-17a,19-dihydroxy- (9,11)-epoxy-4-ene-3,18-dione-20-acetoxy	*Penicillium citrinum* SCSIO 41017	Sponge *Callyspongia* sp	Cytotoxic	[28]
2021	Daldinins G and H	*Penicillium glabrum* glum 003	Soft coral	–	[43]
2021	16α-Hydroxy-17β-methoxy-deoxydihydroisoaustamide, 16β-hydroxy-17α-methoxy-deoxydihydroisoaustamide, 16α-hydroxy-17α-methoxy-deoxydihydroisoaustamide, 16,17-dihydroxydeoxydihydroisoaustamide, 16β,17αdihydroxy-deoxydihydroisoaustamide, 16α,17α-dihydroxy-deoxydihydroisoaustamide and 3β-hydroxy-deoxyisoaustamide	*Penicillium dimorphosporum* KMM 4689	Coral	Statistical increase of PQ-treated Neuro-2a cell viability	[97]
2021	7-Hydroxy-3,10-dehydrocyclopeptine	*Penicillium polonicum* MCCC3A00951	Mangrove	–	[98]
2020	Penicitrinone G	*Penicillium citrinum*	Marine Sponge	Inactive against *M. smegmatis*	[26]
2020	Citridones H–L and ent-Citridone A	*Penicillium* sp. XZD3-3	The gut of a marine shrimp	Anti-inflammatory	[22]
2020	4-Hydroxyscytalone, 4-hydroxy-6-dehydroxyscytalone, demethylcitreoviranol and 4-hydroxy-3,6-dimethyl-2-pyrone	*Penicillium* sp. KMM 4672	Brown algae *Padina* sp	Neuroprotective	[99]
2020	Oxalicine C, penicierythritols A and B	*Penicillium chrysogenum* XNM-12	Marine algae	Antimicrobial	[23]
2020	Chermebilaenes A and B	Co-culture of *P. bilaiae* MA-267 and *P. chermesinum* EN-480	Marine organism	Antimicrobial	[75]
2020	Paspaline, 3-deoxo-4b-deoxypaxilline, 6,7-dehydropaxilline, emindole SB, paspalinine, paspalitrem C, paspalitrem A, penialidin C, and penialidin A	*Penicillium javanicum*	Mangrove rhizosphere soil	Antimicrobial	[100]
2020	Penilactonol A, (2’R)-stachyline B and (2’R)-westerdijkin A	*Penicillium chrysogenum* LD-201810	Marine red alga *Grateloupia turuturu*	Cytotoxic	[52]
2020	Purpurides E–G, peniisocoumarin H	*Penicillium minioluteum* ZZ1657	Marine organism	Antimicrobial	[44]
2020	9-Dehydroxysargassopenilline A and 1,2-didehydropeaurantiogriseol E	*Penicillium cyclopium* SD-413	Deep sea	Antibacterial	[30]
2020	13β-Hydroxy conidiogenone C, 12β-Hydroxy conidiogenone C and 12β-Hydroxy conidiogenone D	*Penicillium* sp. TJ403-2	Sea sediment	Anti-inflammatory	[66]
2020	Penicildiones A − D	*Penicillium* sp. SCSIO41201	Soft coral	Cytotoxic	[83]
2020	Penicacids E − G	*Penicillium parvum* HDN17-478	Marine sediment	Cytotoxic	[101]
2020	Ketidocillinones A–C	*Penicillium* sp. HDN151272	Sponge	Antibacterial	[53]
2020	Notoamide C, cyclotryprostatin E, verruculogen TR-2, citrinin F, isochromophilone V, dehydroaustin and sesquicaranoic acid B	*Penicillium janthinellum*	Marine organism	Antibacterial	[102]
2020	Penoxahydrazones A–C, penoxazolones A and B	*Penicillium oxalicum*	Deep sea cold seep sediments	Antibacterial	[103]
2020	Andrastone C	*Penicillium* sp. SY2107	Marine Sediment	Antimicrobial	[74]
2020	Chermesiterpenoids A–C	*Penicillium chermesinum* EN-480	Marine red alga	Antimicrobial	[60]
2020	Paspaline, 3-deoxo-4b-deoxypaxilline, 6,7-dehydropaxilline, emindole SB, paspalinine, paspalitrem C, paspalitrem A, penialidin C and penialidin A	*Penicillium javanicum* HK1-23	Mangrove rhizosphere soil	Antimicrobial	[100]
2020	Ethyl 8-hydroxyhexylitaconate, methyl 8-hydroxyhexylitaconate and ethyl 9-hydroxyhexylitaconate	*Penicillium antarcticum*	Tunicate *Aplidium pallidum*	Inhibitors of mesenchymal stem cell differentiation	[81]
2020	Neobulgarone D, neobulgarone E, and neobulgarone F	*Penicillium roseopurpureum* (KP1-135C)	Marine alga *Petalonia fascia*	Antimicrobial	[77]
2020	Paraherquamide J	*Penicillium janthinellum* HK1-6	mangrove rhizosphere soil	No antibacterial activity	[19]
2020	5-[2-Hydroxypropane-1-yl]-2,6-dimethlbenzene-1,3-diol and coniochaetone L	*Penicillium* sp. SCSIO 06720	Deep-sea	Antibacterial	[104]
2020	Restricticin B	*Penicillium janthinellum*	Sea sediment	Anti-neuroinflammatory	[78]
2020	Austalide Y 3β-Hydroxy-agathic acid and 3β-Acetoxy-agathic acid	*Penicillium thomii* YPGA3	Deep-Sea	Cytotoxic	[67]

**Table 2:** Docking results of novel and known metabolites isolated from marine-derived *Penicillium* sp

Isolated compound	Enzyme/target	Binding energy (∆G kcal/mol)	Bonding interaction	Activity supporting docking	Refs.
13-Epi-conidiogenone F	Sortase A	− 7.1	Hydrogen-bonding interaction between Glu105 and its OH-group at C-13, and hydrophobic interaction between the keto-group at C1 and Gly192 as well as another hydrophobic interaction with Ala92, Thr93, Thr187, Trp194, and Ala104	Inhibitory activity	[45]
Conidiogenone F		− 6.3	Hydrogen bonding interaction between Arg197 and its OH-group at C-13 and hydrophobic interactions with only Ala104 and Ile182		
4-Hydroxyleptosphin C		− 6.7	Hydrogen-bonding interactions between its keto-group at C-4 and Arg197 and OH-group at C-4 and Glu105 and hydrophobic interactions with Ile199, Ile182		
		− 7.4	Hydrogen-bonding interaction between Arg197 and the keto-group at C-4 and hydrophobic interactions with Ala104, Ile182, Ala92, and Thr93		
Leptosphin C		− 6.4	Hydrogen-bonding interaction between Arg197 and its keto-group at C-13 and hydrophobic interactions with Ile182		
		− 7	No hydrogen-bonding interactions and hydrophobic interactions with Ala92, Gly192, Ile182, and Ala104		
Cyclopiumolides A and B	PARP 1 active sites	–	Cyclopiumolide A was able to engage with PARP 1 by generating key hydrogen bonds with residues Gln-707, Ser-711, Ser714, and Asp-993, while Cyclopiumolide B was by forming hydrogen interactions with four amino acid residues Gly-863, Ser-864, Tyr-896, and Ser-904	Inhibitory activity	[31]
Penicillenol H	SARS-CoV-2 main protease (SARS-CoV-2 Mpro)	– 4.9	Two hydrogen bonds and two intermolecular hydrophobic interactions	Inhibitory activity	[105]
Eutypoid F	Phosphatidylinositol 3- kinase (PI3K)	− 11.6	Phenolic hydroxy groups formed hydrogen bonds with the active site residues THR887, TYR867, and ALA885, and the ester group also interacted with VAL882 by hydrogen bond	Inhibitory activity	[106]
Vridicatol	Peptide deformylases *V. parahaemolyticus*	– 8.2	Gly84 forms a hydrogen bond with the carbonyl oxygen at C-2 position, while Gln47, Leu86, His127 and His131 form four hydrogen bonds with the phenolic hydroxyl at C-3’ position (five H-bonds)	Inhibitory activity	[107]
	*V. cholerae*	– 7.9	Asp42 and Asn43 form two H-bonds with the phenolic hydroxyl at C-3’ position, and Asp96 forms a hydrogen bond with the carbonyl oxygen at C-2 position. Besides, the secondary amine at N-1 position forms two hydrogen bonds with Asp96 and Tyr98		
	*V. vulnifcus*	– 7.9	Asn43 forms two hydrogen bonds with secondary amine at N-1 and alcohol hydroxyl at C-3, while Cys91 and Val94 form two hydrogen bonds with the phenolic hydroxyl at C-3’		
Cyclopenol	Peptide deformylases *V. parahaemolyticus*	– 5.2	2 Hydrogen bonds with TYR106 and ASN68		
	*V. cholerae*	– 7.8	Carbonyl oxygen at C-2 and hydrogen at N-1 form two hydrogen bonds with Gly90, and phenolic hydroxyl at C-13 forms a hydrogen bond with carbonyl oxygen of Glu134		
	*V. vulnifcus*	– 7.5	Gly90 forms an H-bond with amino hydrogen at N-1, and His133, Glu134 and His137 form three H-bonds with phenolic hydroxyl at C-13		
Cyclopenoin	Peptide deformylases *V.parahaemolyticus*	– 5.7	Hydrogen bond with MET1 Amino hydrogen at N-1 and carbonyl at C-2 form two H-bonds with Gly90		
	*V. cholerae*	– 7.8	Three hydrogen bonds with three amino acids (Ile45, Gly46 and Leu92) through C-2 carbonyl and C-3,10 epoxy groups, respectively		
	*V. vulnifcus*	– 7.3	Amino hydrogen at N-1 and carbonyl at C-2 form two H-bonds with Gly90		
Penicillol B	iNOS enzyme	− 7.5	Hydrogen bond with the key amino acid residue GLU-371 through the methoxy group, two hydrogen bonds with the residue ASP-379 and ARG-382 by the hydroxyl group in the iNOS active pocket, respectively	Inhibitory activity	[94]
Asperpendoline	Keap1	− 8.4	Hydrogen bonds with amino acid residues of Val608, Val369, Val418, Val465, and Val467 along with the respective distance of 2.1, 2.2, 1.8, 3.0, and 2.3 Å, and hydrophobically interacting with residues of Cys513, Ala466, and Val420	Inhibitory activity	[108]
Penifuranone A	Inducible nitric oxide synthase (iNOS)	− 6.3	Hydrogen bond with the key amino acid residue ARG-382 through the ester group, three hydrogen bonds with the residue ASP-379, and one hydrogen bond with the residue HEM-901 in the iNOS active pocket	Inhibitory activity	[89]
Steckfusarin A	Superoxide dismutase	− 6.3	Two hydrogen bonds with the residues SER-32 and GLN-153, and five hydrophobic interactions with the residues LYS-4, VAL-6, HIS-20, ALA-152 and GLN-153	Inhibitory activity	[33]
Steckfusarin B		− 6.6	Six hydrogen bonding interactions with the residues GLN-23, ARG-79, ARG-79, SER-102, LEU-103 and ILE-104 and two hydrophobic interactions with LEU-103 and ALA-105 in the active site of superoxide dismutase		
Aceneoherqueinone A	Angiotensin-I-converting enzyme (ACE)	–	Hydrogen interactions with residues Ala261, Gln618, Trp621, and Asn624	Inhibitory activity	[48]
Aceneoherqueinone B			Hydrogen interactions with residues Asp358 and Tyr360		
Pannorin	Monoamine oxidase (MAO)-A	− 25	Hydrogen bonds at Q215 and N181	Inhibitory activity	[109]
	MAO-B	− 24.1	Hydrogen bonds at C172		
Janthinellumine A, Janthinellumine B and Janthinellumine G	Viral neuraminidase	–	They form more than one hydrogen bond with amine of Arg371 side chain in the binding pocket	Inhibitory activity	[65]
Paspalitrem C	PTP1B protein	–	Hydrogen bond with the amino acid residues of GLN-262 and hydrogen interactions with PHE-182, ALA-217 and ILE-219. Also, the 32-CH3 group could form hydrogen bonds with TYR46, VAL-49, and ALA-217. The chain of 21 could form hydrogen interactions with both of ASP-181, PHE-182 and LYS-120	Inhibitory activity	
Shearinine E	TCPTP protein		Two hydrogen bonds were formed between the atoms of O-8/O-25 in 11 and the amino acid residues of ARG524/CYS-532 in the target protein TCPTP and hydrophobic interactions with ASP-548/ARG524		
Shearinine A	SHP-1 protein		Its indole ring and 13-OH formed the hydrogen bonds with ALA-320 and ALA-448 respectively, an π-sulfur interaction with the sulfur atom of LYS-310 and hydrophobic interactions withTHR-322/GLN-422/ GLU-443		
Emindole SB	SHP-2 protein		Its indole ring and 7-OH formed the hydrogen bonds with GLU-249 and THR-253, respectively and Hydrophobic interactions with ARG-111/GLU-250/LEU-233		
Penpaxilloid A	Protein tyrosine phosphatase 1B (PTP1B)	Ki = 5.0 μM	The hydroxyl group of 1 at C-23 formed hydrogen bonds with residues Arg-79 and hydrophobic interactions with residues Phe-196 and Phe-280	Inhibitory activity	[110]
paspalinine-13-ene	α-glucosidase	− 10.9	Two hydrogen bonds with Gln279 and His280 and hydrophobic interactions with residues Tyr158, Phe178, Val216, and Arg315		
Bialorastin C	Vascular endothelial growth factor receptor 2 (VEGFR2)	–	Five hydrogen bonds with residues of VEGFR2, including three hydrogen bonds between lactone bridge and residues ASN-923, THR-926, and ARG-929, one hydrogen bond between oxygen bridge and residue ARG-1032, and one hydrogen bond between oxygen atom of ketone C-17 and residue ARG1080	Activating activity	[111]
JanthinellumineJ	TCPTP	–	Hydrogen bonds with SER528 of protein, ARG-754, GLY-759, GLN-762, ASP-548 and VAL-549 and hydrophobic interactions with multiple residues of protein (GLU249 and GLU-250, THR-253, PRO-215, and PRO-491)	Inhibitory activity	[112]
JanthinellumineK	SHP2		Hydrogen bond with THR-253, PHE-113, HIS-114, THR-218, ARG-229 and SER-228, π-π stacking interactions with HIS-114 and hydrophobic interactions with PRO-446 and GLU-443		

## Conclusion

This study shed the light on the recent discovery of bioactive MNPs (marine-derived *Penicillium*; natural products) derived from *Penicillium* fungi found in marine environments in the last five years from 2020 to 2025, classified the MNPs based on the sources of the fungi and their specific biological activities. Besides, the results of molecular docking studies recently performed on *Penicillium* metabolites referring to various biological activities were also compiled in this review.

## Data Availability

Data availability is not applicable.
